# Predictors of physicians’ stress related to information systems: a nine-year follow-up survey study

**DOI:** 10.1186/s12913-018-3094-x

**Published:** 2018-04-13

**Authors:** Tarja Heponiemi, Hannele Hyppönen, Sari Kujala, Anna-Mari Aalto, Tuulikki Vehko, Jukka Vänskä, Marko Elovainio

**Affiliations:** 1 National Institute for Health and Welfare, P.O. Box 30, 00271 Helsinki, Finland; 2 Aalto University, Espoo, Finland; 3 Finnish Medical Association, Helsinki, Finland; 4 University of Helsinki, Helsinki, Finland

**Keywords:** Health information systems, Physicians, Stress, Electronic health records, Cognitive workload

## Abstract

**Background:**

Among the important stress factors for physicians nowadays are poorly functioning, time consuming and inadequate information systems. The present study examined the predictors of physicians’ stress related to information systems (SRIS) among Finnish physicians. The examined predictors were cognitive workload, staffing problems, time pressure, problems in teamwork and job satisfaction, adjusted for baseline levels of SRIS, age, gender and employment sector.

**Methods:**

The study has a follow-up design with two survey data collection waves, one in 2006 and one in 2015, based on a random sample of Finnish physicians was used. The present study used a sample that included 1109 physicians (61.9% women; mean age in 2015 was 54.5; range 34–72) who provided data on the SRIS in both waves. The effects of a) predictor variable levels in 2006 on SRIS in 2015 and b) the change in the predictor variables from 2006 to 2015 on SRIS in 2015 were analysed with linear regression analyses.

**Results:**

Regression analyses showed that the higher level of cognitive workload in 2006 significantly predicted higher level of SRIS in 2015 (β = 0.08). The reciprocity of this association was tested with cross-lagged structural equation model analyses which showed that the direction of the association was from cognitive workload to SRIS, not from SRIS to cognitive workload. Moreover, increases in time pressure (β = 0.16) and problems in teamwork (β = 0.10) were associated with higher levels of SRIS in 2015, whereas job satisfaction increase was associated with lower SRIS (β = − 0.06).

**Conclusions:**

According to our results, physicians’ cognitive workload may have long-lasting negative ramifications in regard to how stressful physicians experience their health information systems to be. Thus, organisations should pay attention to physicians workload if they wish physicians to master all the systems they need to use. It is also important to provide physicians with enough time and collegial support in their system-related problems, and in learning new systems and system updates.

## Background

Poorly functioning, time consuming and inadequate information systems (IS) have emerged among the important stress factors in physicians’ work [1–3]. In addition, it seems that this stress keeps increasing [2]. In 2010 and 2014, Finnish physicians in various working sectors evaluated their electronic health record (EHR) systems very critically [4, 5]. On a scale from 1 (*fail*) to 7 (*excellent*), the average ratings varied from 3.2 to 4.4 in 2014 [5]. The problems associated with IS may have negative ramifications for patient care, such as problems in clinical performance and patient safety [6].

Usability problems and deficiencies – such as system failures, slowness, a lack of integration, poor support for documentation and poor retrieval of patient data from other organisations – are among the most prominent problems in IS reported by physicians [1, 4, 7–9]. Poor usability and other deficiencies have been associated with physicians’ stress and professional dissatisfaction [10, 11]. The use of IS has also changed the traditional doctor–patient relationship and physicians spend more time interacting with computers than with clients [12–14], which may also be frustrating for physicians.

There may also be other factors that may increase the frustration and stress about IS. Changes in IS require physicians to constantly develop their skills. In Finland it has been found that learning to use the EHR requires a lot of training, and physicians experience that training needs have increased between the years 2010 and 2014 [15]. However, the time pressures of care and inadequate staffing levels may not allow enough time to learn to master all the complex functions of the systems [16]. It is possible that a lack of time to learn to use all the new systems and functions leads physicians to regard IS as being extremely complicated and stressful. Indeed, time pressure has been found to be related to negative outcomes – such as burnout, dissatisfaction, and intent to leave – among those physicians who had high number of EHR functions [10].

Physicians’ work includes complex and demanding activities such as multitasking, clinical reasoning, problem-solving, and a need to deal with vast amounts of information [6, 17, 18]. All of these may cause cognitive workload according to Kirsch [19], who has identified too much information supply, too much information demand, constant multitasking and interruptions as examples of causes of cognitive overload in the workplace. Information chaos theory [6], conceptualizes five information hazards: information overload, underload, scatter, conflict, and erroneous information as information chaos. These hazards are experienced by physicians on a daily basis and can together or separately increase the risk of information-related errors. Information overload occurs when there is too much data for a physician to organize, synthesize, act, or draw conclusion from. EHRs may make the information overload situation worse by encouraging electronic copying and pasting, adding irrelevant information and mixing data. Information scatter occurs when information is located in multiple places and EHRs may worsen this, for example because of inadequate search methods and multiple windows. A high workload may result in a situation where physicians have less resources and capacities to cope with difficult IS and hence they experience them as more stressful. In addition to direct workload, other factors may also impact on how stressful IS are experienced to be. For example, poor work relationships, which have been associated with stressful experiences [20], may cause a person to get less collegiate support and further diminish their tolerance of IS-related stress. Support at work has been suggested as a possible buffer for the effect of high work strain on stress-related illness [21]. Moreover, positive attitudes, such as job satisfaction [22, 23], may improve tolerance towards demanding and frustrating situations involving IS.

Finland is a suitable country for studying the stress that comes from IS, given that there have been multiple reforms in Finland lately regarding IS in the health care sector. The EHR adoption in public and private sectors in Finland is high reaching 100% in 2010 regarding the public sector [24]. The national digital repository for electronic patient data (called Kanta) has been launched in Finland (during 2012–2017), which is targeted to health care service providers, pharmacies and citizens. Kanta provides services such as electronic prescriptions, My Kanta pages for citizens, a patient data repository and a pharmaceutical database. Health care providers using electronic documentation have to join Kanta and with one exception all the pharmacies and public service providers had joined the national e-prescription service by the end of 2014 [24]. Also, substantial amount of the private sector providers used e-prescription at that time.

It seems that many factors – also those not directly linked to IS – may influence the tolerance of IS-related problems, and by identifying and improving those factors, the factors negativity associated with IS might decrease. The present nine-year longitudinal study aimed to examine the predictors of physicians’ stress related to information systems (SRIS) among Finnish physicians. The examined predictors were cognitive workload, staffing problems, time pressure, problems in teamwork and job satisfaction, adjusted for baseline levels of SRIS, age, gender, and employment sector. We questioned a) whether the levels of these predictor variables were associated with the levels of SRIS nine years later and b) whether changes in these predictor variables from the year 2006 to 2015 were associated with SRIS in 2015.

## Methods

### The study sample

The present study gathered a random sample of 5000 physicians in Finland (30% of the whole physician population) in 2006 as a part of the Finnish Health Care Professionals Study. The data was gathered from Finnish Medical Association’s register which covered all licensed physicians in Finland at that time. In 2006 postal questionnaires were sent to the physicians with two reminders to non-respondents and 2841 physicians responded to the questionnaire (response rate: 57%). Regarding age, gender and employment sector the sample corresponds to the eligible population [25]. Ethical approval for the study was obtained from National Institute for Health and Welfare.

The follow-up data was gathered in 2015 by using either a web-based questionnaire or a traditional postal questionnaire. In 2006 the respondents were asked their consent to future follow-up and 2206 agreed. 47 physicians were omitted because they had died or address was unknown, thus the questionnaire was sent to 2159 physicians in 2015. Of these 1462 physicians responded (response rate 68.3%). The present sample consisted of those 1109 physicians (61.9% women; mean age in 2015 was 54.5; SD = 9.1; range 34–72) who had answered the SRIS variable in both data gathering times. Women (57.4% in the eligible population) and older respondents (mean age of the eligible population: 47.3) are slightly more represented in the present sample than in the eligible population. Due to missing information for some variables, *n* varied between 1109 and 1009 in analyses.

### Measurements

*SRIS* was measured with the mean of two items, framed in one question asking how often (during the past half-year period) the respondent had been distracted by, worried about, or stressed about: a) constantly changing information systems and b) difficult, poorly performing IT equipment/software. The answers were rated on a five-point Likert scale ranging from 1 (*never*) to 5 (*very often*). The scale’s reliability (Cronbach’s alpha) was 0.84 in 2006 and 0.85 in 2015 in the present sample. This measure has previously been used and associated with employees’ distress (General Health Questionnaire) and higher levels of on-call duties [26, 27].

*Cognitive workload* in 2006 was measured with four items (α = 0.70) measuring how often (during the past half-year period) a person had been distracted by, worried about or stressed about 1) the need to continually do complex problem-solving, 2) extensive and changing expertise needs, 3) responsibility for patients and 4) constant interruptions and difficulties in completing tasks. The items were rated on a five-point Likert scale ranging from 1 (*never*) to 5 (*very often*). A mean value of the four items was calculated, bigger values indicating higher cognitive workload. In 2015, we assessed only one of these items (namely *constant interruptions and difficulties in completing tasks*) thus the change score from the years 2006 and 2015 was calculated using only that item.

*Staffing problems* were measured with a mean of four items (α = 0.70) measuring how often (during the past half-year period) a person had been distracted by, worried about or stressed about 1) the inadequacy of physicians, 2) the inadequacy of other staff, 3) the uneven segmentation of workload amongst personnel and 4) changing physicians and short-time temps. The items were rated on a five-point Likert scale ranging from 1 (*never*) to 5 (*very often*), higher scores indicated more problems. In 2015 we assessed only one of these items, namely *the inadequacy of physicians*, thus the change score from the years 2006 and 2015 was calculated using only that item.

*Time pressure* was measured with the mean of three items (α in 2006 = 0.84 and in 2018 = 0.87) measuring how often (during the past half-year period) a person had been distracted by, worried about or stressed about 1) being in a constant hurry and time pressure coming from unfinished work tasks, 2) having too little time to do work properly and 3) the forced pace of work. The items were rated on a five-point Likert scale ranging from 1 (*never*) to 5 (*very often*). This measure has been widely used previously and associated with on-call duties and strain among physicians [26, 28].

*Problems in teamwork* were measured with four items (α = 0.76) measuring how often (during the past half-year period) a person had been distracted by, worried about or stressed about 1) problems in human relationships at work, 2) a lack of trust and openness in the workplace, 3) a lack of co-operation in the work unit and 4) pressure for conformity in the work unit. The items were rated on a five-point Likert scale ranging from 1 (*never*) to 5 (*very often*) and higher scores indicated higher problems. This measure has previously been associated with physicians’ distress, work ability and self-rated health [27].

*Job satisfaction* was assessed with the mean of three items (α = 0.68; e.g., *I am generally satisfied with my work*) derived from Hackman and Oldham’s [29] Job Diagnostic Survey on a five-point scale ranging from 1 (*totally disagree*) to 5 (*totally agree*).

*Employment sector* was categorized into four groups: primary care, hospitals, the private sector, and other sectors.

### Statistical analysis

The effects of predictor variable levels in 2006 on SRIS in 2015 were analysed with linear regression analyses. The analyses were conducted in three steps. In the first step, the analyses included baseline levels of SRIS (2006), age, gender, employment sector, cognitive workload and staffing problems. In the second step, time pressure and problems in teamwork were added to the former model. Finally, also job satisfaction was additionally added. The analyses were conducted in these steps to find out a) whether time pressure or problems in teamwork would partly account for the possible effects of cognitive workload or staffing problems on SRIS and b) whether job satisfaction would partly account for the possible effects of all previously mentioned variables on SRIS.

We tested the reciprocal associations between SRIS and other psychosocial factors using cross-lagged structural equation modelling (SEM). In all SEM models, the contribution of the potential confounding factors in the relationships between SRIS and other psychosocial factors were taken into account by using adjusted values (adjusted for age, gender and employment sector), predicted by the linear regression models. The cross-lagged analyses were applied to all available data for individuals who responded during either of the data collection phases using maximum likelihood (ML). The direction for the associations was evaluated by a) evaluating the significance of the associations and b) comparing the fit of the models where either direction of the associations were dropped to the saturated model (where both directions were present). We evaluated the goodness-of-fit of the models using multiple fit indices: chi-square, the root mean square error of approximation (RMSEA), the Akaike information criterion (AIC), the Bayesian information criterion (BIC), the comparative fit index (CFI), and the Tucker-Lewis Fit Index (TFI). A non-significant chi-square value suggests good model fit. Chi-square is, however, highly sensitive to sample size. RMSEA values of less than 0.05 and 0.10 represent a good and acceptable fit, whereas CFI values above 0.90 and 0.95 indicate an acceptable and good fit [30]. In comparing alternative models, a statistically significant improvement in the chi-square value indicated an improved model fit. Complete scales (not the items) were used in the SEM models. These analyses were performed using the Lavaan R-package (version 0.5–23.1097).

We also examined the effects of the change of predictor variables on SRIS in 2015 with linear regression analyses. Change scores were calculated by subtracting the 2006 scores from the 2015 scores. These analyses were conducted in similar steps to those of the analyses with predictor variable levels in 2006. All analyses were performed using SPSS software version 24.0.

## Results

The characteristics of the study population can be seen in Table 1.

**Table 1:** The characteristics of the study sample

	2006		2015	
	n	%	n	%
Sector				
Hospital	489	44.5	446	40.2
Primary care	243	22.2	219	19.8
Private	134	12.2	244	22.0
Other	232	21.1	199	18.0
	Mean	SD	Mean	SD
SRIS	2.93	1.2	3.48	1.1
Cognitive workload	2.61	0.8	2.89	1.24
Staffing problems	2.84	0.9	3.02	4.4
Time pressure	3.37	1.0	3.05	1.1
Problems in team work	2.17	0.8	2.10	0.8
Job satisfaction	5.43	1.1	4.10	0.8

The scale varied between 1 and 5 for all other continuous variables except for job satisfaction the scale ranged between 1 and 7

**Fig. 1:**
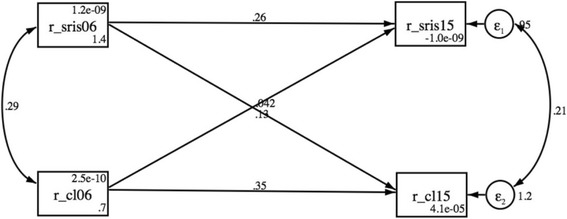
Cross-lagged SEM model between stress related to information system (r_sris) and cognitive load (r_cl)

Table 2 shows the results of regression analyses regarding the levels of variables in 2006 as predictors of SRIS in 2015 when the baseline levels of SRIS (2006), age, gender and employment sector were adjusted for. The higher level of cognitive workload in 2006 was significantly associated with higher level of SRIS in 2015. This significant association remained after all adjustments. However, the explanatory power of the other variables than baseline level and demographics seemed rather low, given that the increase of R^2^ was low in Steps 2 and 3. We additionally tested the reciprocity of cognitive workload-SRIS association with cross-lagged structural equation models using the complete scales. The repeated cross-lagged SEM was only conducted for cognitive workload. As can be seen in Fig. 1, cognitive workload in 2006 predicted SRIS in 2015 (βeta = 0.13; *p* = 0.001), but SRIS in 2006 did not predict cognitive workload in 2015 (βeta = 0.04; *p* = 0.155) in addition to autoregressive associations (the model tested both directions simultaneously). Comparing the model fit indexes (Table 3) also showed that it was possible to drop the pathway from SRIS 2015 to cognitive workload in 2015 without significantly reducing the fit of the model (**Δ**χ^2^ = 2.32; *p* = 0.128) compared to the saturated model. In contrast, it was not possible to drop the pathway from cognitive work load to SRIS 2006 without significantly reducing the fit of the model (**Δ**χ^2^ = 7.89; *p* = 0.005).

**Table 2:** The association of predictor variables (2006) with stress related to information systems in 2015. The results from regression analyses

	Step 1^a^			Step 2^a^			Step 3^a^		
	t	p	β	t	p	β	t	p	β
Cognitive workload	2.23	0.026*	0.07	2.20	0.029*	0.08	2.21	0.027*	0.08
Staffing problems	1.43	0.154	0.05	1.73	0.085	0.07	1.78	0.076	0.07
Time pressure				−0.12	0.907	− 0.01	0.04	0.965	0.00
Problems in team work				−1.57	0.118	−0.05	−1.22	0.222	−0.04
Job satisfaction							1.15	0.251	0.04
R^2^	0.17			0.18			0.18		

^a^All analyses were adjusted for baseline level, age, gender, and employment sector

**Table 3:** Fit indices of the cross-lagged SEM model of the associations between stress related to information systems (SRIS) and cognitive load

	DF	AIC /BIC	RMSEA	CFI/ TFI	**Δ**χ^2^	*p*-value for difference
Saturated model	0	11,962 / 11,997				
Only from SRIS to cognitive load	1	11,962 / 11,992	0.077	0.97/ 0.87	7.89	0.005**
Only from cognitive load to SRIS	1	11,967 / 11,997	0.035	0.99/ 0.97	2.23	0.128

RMSEA should be < 0.05 and CFI/TFI > 0.90

Table 4 shows the results of regression analyses regarding the effect of the changes of variables between 2006 and 2015 on SRIS in 2015 when the baseline levels of SRIS (2006), age, gender and employment sector were adjusted for. An increase in staffing problems was significantly associated with higher SRIS, but this association did not remain after adjusting for changes in time pressure and problems in teamwork. A *t*ime pressure increase and a problems in teamwork increase were associated with higher SRIS, whereas a job satisfaction increase was associated with lower SRIS.

**Table 4:** The association of changes in predictor variables (from 2006 to 2015) with stress related to information systems in 2015. The results from regression analyses

	Step 1^a^			Step 2^a^			Step 3^a^		
	t	p	β	t	p	β	t	p	β
Cognitive workload change	1.25	0.212	0.04	−0.80	0.422	−0.03	−0.62	0.537	−0.02
Staffing problems change	2.25	0.025*	0.07	−0.64	0.524	−0.02	−0.43	0.667	−0.01
Time pressure change				4.91	< 0.001***	0.17	4.37	< 0.001***	0.16
Problems in team work change				4.03	< 0.001***	0.12	3.04	0.002**	0.10
Job satisfaction change							−2.00	0.046*	−0.06
R^2^	0.17			0.21			0.21		

^a^All analyses were adjusted for baseline level, age, gender, and employment sector

## Discussion

According to our results, cognitive workload predicts stress related to difficult, poorly performing and constantly changing IS in our nine-year follow-up. Moreover, an increase in time pressure and in problems in teamwork predicted higher levels of this stress, whereas an increase in job satisfaction predicted lower levels. Our findings are congruent with previous findings showing the negative effects of cognitive workload and poor work relationships on well-being and stress experiences [19, 20] and the positive effects of job satisfaction on stress [22, 23]. Correspondingly, previous findings have shown the associations of time pressure with burnout, dissatisfaction and intent to leave [10]. It has also been shown that ICT-related demands are associated with higher strain, stress and burnout [31].

Too much information, constant multitasking and interruptions may cause cognitive overload in physicians, which in turn may lead to long-term problems in coping with the difficulties and challenges resulting from non-functional IS. It has been shown that only selective and reduced capacity functions are carried out when people have a high cognitive and mental workload [32], thus learning new systems may be difficult at those times. This is worrying, given that multitasking, clinical reasoning, problem-solving and a need to deal with vast amounts of information are common daily routines among physicians and they also can have negative consequences for physician performance and patient safety [6, 17, 18].

We showed that the increase in time pressure from 2006 to 2015 predicted higher levels of SRIS in 2015. Systems change often and physicians have to learn to master the new systems and are required to constantly develop their skills. In Finland, it has been found that learning to use EHR requires a lot of training, and the time needed for this learning has increased between the years 2010 and 2014 [15]. However, both the time pressures of care and inadequate staffing levels may limit the time/capacity to learn to master all the complex functions of the systems [16]. It is possible that a lack of time to learn to use all the new systems and functions may lead physicians to regard IS as extremely complicated and stressful.

Our findings suggest that when the time allocated does not correspond to the time needed to provide high quality care, it may challenge coping with a demanding IS and lead to negative ramifications. Time has been suggested as perhaps the most important resource when dealing with information chaos, and if information chaos occurs in an environment with time constraints (such as scheduled 15-min appointments for patients with multiple problems), the impact on physicians is exacerbated [6]. Time pressure during office visits and examinations has been associated more strongly with physicians’ burnout, dissatisfaction and intent to leave among those physicians who had a high number of EHR functions compared to those with a low number of functions [10]. Visit preparation, appropriate practice redesign and well-designed EHRs have been suggested to decrease time problems and information chaos during patient visits [6].

EHRs may reduce the time required for prescribing and communicating between professionals within the working organisation, but they may also increase time needed for patient documentation, chronic disease management and preventive care tasks [33]. Boonstra and Broekhuis [34] stated in their review that EHRs are likely to slow physicians workflow because they require additional time to select, implement, enter data and learn how to use them, which is likely to lead to reduced productivity and increased workload. However, there also exist opposite findings showing that EHR use is associated with higher productivity [35, 36].

We also found that an increase in problems in teamwork predicted higher levels of SRIS. Previous studies have also highlighted teamwork and co-operation in connection with IS. Physicians work in co-operation with other health care professionals and they need support from colleagues, other professionals and management in order to learn and master an IS [34]. Expert support (which refers to assistance from one physician to another) has been found to be critical for the adoption of EHRs [37]. Good communication and team spirit help co-workers to share their experiences and solutions when problems with systems arise and more experienced/advanced users can advise users with less experience. Communication among users has been found important for user acceptance of EHRs, and it has been shown that people need support for co-operation within a team in order to facilitate the adoption of new systems [37]. However, ICT systems have been criticized for not promoting physician–nurse collaboration, cross-organizational collaboration or physician–patient collaboration [4].

Besides finding factors that predispose physicians to higher levels of SRIS, we also found that an overall job satisfaction increase predicted lower levels of SRIS. Thus, a physician who is satisfied with his or her job is also more prone to experience IS as less stressful. This is congruent with previous finding that job satisfaction has been associated with EHR satisfaction [38]. It has been shown that physicians’ job satisfaction can be increased by proficiency training [39].

The present study relied on self-reported measures, which may lead to problems associated with an inflation of the strengths of relationships and with common method variance. To minimise problems with self-reports we used measures that showed good reliability. However, even though many of our measures have been widely used in scientific articles many of our instruments have not been specifically validated in proper validation studies. Our results may have been affected because there was some overlap in our predictor variables and outcome, given that many of our variables dealt with the experience of stress (coming from different bases). One limitation of our study is that we did not measure all the items from both years, for example, we did not assess all the items from cognitive workload and staffing levels in 2015 that were assessed in 2006. Therefore, our finding that associations of increases in cognitive workload and staffing level with SRIS were not significant should be taken cautiously. Future studies should examine this in more detail. Moreover, although we controlled for many factors – such as age, gender and employment sector – we cannot rule out the possibility of residual confounding. In addition, our sample is not totally representative of the present physician population in Finland. Our sample included a higher percentage of women than the mean percentage for the eligible population. Moreover, because our sample was gathered as a random sample in 2006 our sample also included older physicians and more specialists in 2015 compared to the eligible population in 2015. Due to sample loss our sample may be biased and this may have led to an underestimation of effects for a number of reasons, for example, because highly stressed physicians may have a higher probability of dropping from the sample.

In Finland tax-financed universal health care is provided for all residents, therefore generalizing our findings to countries with other types of health care systems or IT systems should be done with caution. However, digitalisation is increasing at a high pace in most developed countries, thus we may assume that SRIS is also a problem in other developed countries given that physicians from all these countries have to face new challenges coming from IS. A lot has happened regarding digitalisation in Finland between the study years 2006 and 2015. For example, Kanta, the national digital repository for electronic patient data, has been launched, almost all physicians now use EHRs and e-prescription is mandatory. Thus, the burden associated with IS may have changed substantially, which may have had an effect on our results.

## Conclusions

We found that baseline cognitive workload predicted how stressful physicians experienced their IS to be nine years later. Moreover, a nine-year increase in time pressure and in problems in teamwork predicted higher levels of SRIS, whereas a job satisfaction increase predicted lower levels of SRIS. According to our results, work burden and cognitive workload should be taken into account when designing IS and work procedures related to them. It would be good if IS would not additionally increase the work burden of physicians. It has been suggested that interfaces should be designed in a way that they would not negatively affect the cognitive workload experienced by healthcare professionals but instead they would ease the user in completing tasks [40]. According to our results, time is of great importance in relation to IS use. Physicians should be given enough time to learn to master new systems and system updates, as well as being given time to learn how to process electronic patient data. Moreover, good team spirit, social support and overall job satisfaction are important. Physicians need support in their IS-related problems. For example, clerical support personnel for physician order entry has been found to lessen the stress and fatigue after implementing new systems [41]. Future studies should examine more specifically the role of IS in physicians’ cognitive workload and physicians’ IS-related competences.

It has been suggested that EHRs are mainly designed based on the needs of documenting and billing instead of taking better account of the needs of doctors and patients [42]. Moreover, decisions about IS have been shown to be based more on the preferences of IT professionals and hospital administrators than on the preferences of end users, because vendors perceive IT staff and administrators more clearly as the buyers of their systems and give their needs higher priority [43]. Physicians should be included more in the development of systems. It has been shown that physicians are interested in participating in IT systems development [44] and physician-driven improvements to EHR systems have been found to be useful [45].

## Abbreviations

EHR: Electronic health record
IS: Information systems
SRIS: Stress related to information systems
